# Infection par le SARS-CoV-2 chez les femmes enceintes: série tunisienne de 11 cas

**DOI:** 10.11604/pamj.supp.2020.37.1.27185

**Published:** 2020-12-17

**Authors:** Chawki Mrazguia, Haithem Aloui, Hadhami Jaouad, Farouk Jaouad

**Affiliations:** 1 La Rabta, Faculté de Médecine de Tunis, Tunis, Tunisie,; 2 Nabeul Centre, Hôpital Mohamed Tlatli, Nabeul, Tunisie,; 3 Monastir Centre, Centre de Maternité et de Néonatologie de Monastir, Monastir, Tunisie

**Keywords:** Coronavirus, transmission, comorbidité, grossesse, Coronavirus, transmission, comorbidity, pregnancy

## Abstract

**Introduction:**

Chez la femme enceinte, les symptômes de COVID-19 rejoignent ceux de la population générale dans la plupart des cas. Ceci dit comme toute population à risque des symptômes plus graves peut apparaître tels que la pneumonie ou le SDRA. Nous nous proposons dans notre étude de définir les caractéristiques cliniques, biologiques et thérapeutiques ainsi que la prise en charge d´une série de femmes enceintes atteintes de COVID-19.

**Méthodes:**

il s´agit d´une étude rétrospective et descriptive qui a colligée 11 patientes enceintes et atteinte par le Coronavirus SARS-CoV-2 dans le service de gynécologie-obstétrique de l´hôpital Mohamed Tlatli, Nabeul, Tunisie entre le 12 septembre et 11 novembre 2020.

**Résultats:**

la moyenne d´âge est de 31,18 ans. Six patientes étaient suivies pour une maladie chronique. Le diabète gestationnel était la comorbidité la plus fréquente. Un contact avec un cas suspect ou confirmé de COVID-19 était identifié chez 6 patientes .La durée moyenne d´incubation du virus était de 5,89 jours. Le terme moyen de grossesse était de 33,36 semaines d´aménorrhées. La voie d´accouchement privilégiée était la césarienne. Les symptômes les plus fréquemment rapportés étaient la fièvre chez toutes les patientes, la toux sèche, la dyspnée et la céphalée chez neuf patientes. Deux patientes étaient prises en charge ultérieurement en réanimation médicale pour un tableau de syndrome de détresse respiratoire aigüe (SDRA). Les deux patientes étaient mises sous oxygénothérapie à haut débit nasal : l´Optiflow sans qu´il y ait recours à la ventilation mécanique. Dans les 9 autres cas, le traitement symptomatique et l´antibiothérapie avaient suffi et les patientes sont retournées à leur domicile. Nous n´avons eu aucune transmission materno-fœtale et aucun cas de décès. L´allaitement ne semble pas influencer cette transmission.

**Conclusion:**

les symptômes les plus fréquemment rapportés étaient la fièvre chez toutes les patientes, en deuxième lieu la toux sèche, la dyspnée et la céphalée. Il n´y a pas eu de transmission materno-fœtale, ni de transmission au cours de l´allaitement. En général le traitement symptomatique et à base d´antibiothérapie avait suffi. Nous faisons actuellement face à la deuxième vague du virus. Nos efforts devront se baser sur une meilleure connaissance clinique de ce nouveau coronavirus et adapter une conduite en fonction.

## Abstract (en)

**Introduction:**

in pregnant women, the symptoms of COVID-19 join those of the general population in most cases. However, like any population at risk, more serious symptoms can appear such as pneumonia or ARDS. In our study, we propose to define the clinical, biological and therapeutic characteristics as well as the management of a series of pregnant women suffering from COVID-19.

**Methods:**

this is a prospective and descriptive study which brought together 11 pregnant patients affected by the Coronavirus SARS-CoV-2 in the gynecology-obstetrics department of Mohamed Tlatli hospital, Nabeul, Tunisia between 12^th^ September and 11^th^ November 2020.

**Results:**

the average age is 31.18 years. Six patients were followed for a chronic disease. Gestational diabetes was the most common comorbidity. Contact with a suspected or confirmed case of COVID-19 was identified in 6 patients. The average incubation period of the virus was 5.89 days. Average term of pregnancy was 33.36 weeks with amenorrhea. The preferred route of delivery was cesarean section. The most frequently reported symptoms were fever in all patients, dry cough, dyspnea and headache in nine patients. Two patients were subsequently treated in medical intensive care for a picture of acute respiratory distress syndrome (ARDS). Both patients were put on high flow nasal oxygen therapy: Optiflow without the need for mechanical ventilation. In the remaining 9 cases, symptomatic treatment and antibiotic therapy were sufficient and the patients returned to their homes. We have had no mother-to-child transmission and no death. Breastfeeding does not seem to influence this transmission.

**Conclusion:**

the most frequently reported symptoms were fever in all patients, followed by dry cough, dyspnea and headache. There was no mother-to-child transmission, nor transmission during breastfeeding. In general, symptomatic and antibiotic treatment was sufficient. We are currently facing the second wave of the virus. Our efforts will have to be based on a better clinical knowledge of this new coronavirus and adapt a management function accordingly.

## Introduction

Le coronavirus (SARS-CoV-2) cause la maladie COVID-19 [1]. Ce nouveau coronavirus contient une identité nucléotidique similaire à celle du SARS-CoV-2 [2]. La situation est devenue une urgence mondiale le 30 janvier 2020, selon l´OMS [3] et une pandémie à dater du 11/3/2020. D´après le compte du Johns Hopkins Coronavirus Resource Center, il y a, au 11 novembre 2020, en Tunisie, 72993 cas détectés de coronavirus et 2006 décès [4]. La revue de la littérature, bien que limitée chez la femme enceinte, montre que les symptômes rejoignent ceux de la population générale dans la plupart des cas. Ceci dit comme toute population à risque des symptômes plus graves peuvent apparaître tels que la pneumonie ou le SDRA [5]. En effet, les changements physiologiques liés à la grossesse [6] et les changements cardio-pulmonaires pourraient causer une forme grave de pneumopathie [7]. Nous nous proposons dans notre étude de définir les caractéristiques cliniques, biologiques et thérapeutiques ainsi que la prise en charge d´une série de femmes enceintes atteintes de COVID-19.

## Méthodes

Il s´agit d´une étude rétrospective et descriptive qui a inclus tous les patientes prises en charge pour une infection au Coronavirus SARS-CoV-2 dans le service de gynécologie-obstétrique de l´hôpital Mohamed Tlatli, Nabeul, Tunisie entre le 12 septembre et le 11 novembre 2020. Les patientes étaient hospitalisées à partir de leur domicile par le SAMU. Notre protocole thérapeutique associait: i) Pour les patientes non graves ne nécessitant pas une admission en milieu de réanimation: a) Azithromycine 500 mg par jour pendant 5 jours b) Vit C 2 à 3g par jour tout au long de la période d´hospitalisation c) Vit D, une demi ampoule: prise unique d) Zinc 15 mg par jour tout au long la période de l´hospitalisation d) HBPM (héparine de bas poids moléculaire) selon de la gravité en dehors des contre-indications: dose préventive 4000UI/jour avec surveillance bihebdomadaire du taux des plaquettes. ii) Pour les patientes admises en milieu de réanimation: les patientes ont nécessité en plus de l´oxygénothérapie nasale, humidifiée et réchauffée, à haut débit: l´Optiflow dont les paramètres sont optimisés selon les besoins des patientes. Nous avons mis une HBPM à dose curative de 100UI/Kg par jour pendant 3 mois avec une surveillance bihebdomadaire du taux des plaquettes amoxicilline + acide clavulanique 1g * 3/j pendant 8 jours et dexaméthasone 24mg/j. Un consentement éclairé et écrit a été obtenu des patientes avant l´introduction du traitement. Les critères de sortie de l´hôpital étaient une: SpO_2_> 95% à l´air ambiant, l´absence de décompensation des tares associées et la possibilité d´auto-isolement pendant 14 jours après la sortie. Toutes les patientes ont bénéficié d´un contrôle clinique au décours de leur période d´auto isolement.

## Résultats

**Population de l´étude:** onze patientes ont été colligées. Nos patientes étaient âgées entre 22 et 42 ans. La moyenne d´âge est de 31,18 ans. Une patiente était tabagique. Sept femmes étaient des femmes au foyer sans profession. La moyenne d´IMC (indice masse corporelle) était de 28,18 avec des extrêmes allant de 24 à 32. Six patientes étaient suivies pour une maladie chronique .Le diabète gestationnel était la comorbidité la plus fréquente, présente chez cinq patientes. L´hypertension artérielle était présente chez quatre patientes.

**Transmission:** un contact avec un cas suspect ou confirmé de COVID-19 était identifié chez six patientes. Il s´agissait d´un membre de la famille dans quatre cas et une patiente hospitalisée dans la même chambre dans deux cas. Une patiente revenant d´un voyage de la France dans un cas. Aucune exposition à risque n´a été signalée dans quatre cas. La durée moyenne d´incubation du virus était de 5.89 jours (5-7 jours).

**Personnels contaminés:** malgré les moyens de protection (Le masque de protection respiratoire individuelle de type FFP2, visière, gants et blouse jetables pour tous les soins), douze personnes travaillant au service ont été contaminés. Ils étaient en contact avec six de nos patientes. Nous serons tentés de supposer une contamination quoique la possibilité d´une contamination extérieure existe.

**Clinique:** le délai moyen entre l´installation des symptômes et l´hospitalisation était de 6,55 jours (0-12 jours) dont deux patientes déjà hospitalisées, contaminées pendant leur séjour à l´hôpital. Les symptômes les plus fréquemment rapportés étaient la fièvre chez toutes les patientes, la toux sèche, la dyspnée L´asthénie et la céphalée chez 9/11 comme détaillé dans le Tableau 1. La fièvre était objectivée à l´admission chez 6/11 patients.

**Tableau 1:** signes cliniques objectivés chez les femmes enceintes atteintes de COVID-19

Signes cliniques	Nombre de patientes atteintes (N=11)
Fièvre	11
Toux sèche	9
Dyspnée	9
Asthénie	9
Céphalées	9
Vomissements	5
Anosmie et agueusie	5
Diarrhée	2
Odynophagie	1

**Biologie:** le taux d´hémoglobine ainsi que le taux des plaquettes étaient dans les normes chez toutes les patientes. Une insuffisance rénale fonctionnelle était observée chez une patiente. Trois patientes avaient une cytolyse hépatique entre 2 et 3 fois la normale. La protéine C-réactive (CRP) était élevée dans neuf cas.

**Prélèvement virologique:** toutes les patientes ont eu un écouvillonnage naso-pharyngé adressé pour recherche du coronavirus par réaction en chaine par polymérase (PCR). L´analyse virologique était positive chez les 11 patientes.

**Imagerie thoracique:** aucune de nos patientes n´a bénéficié d´une imagerie thoracique vu le contexte de grossesse.

**Complications de la grossesse:** le terme actuel de grossesse (TAG) moyen était 33,36 avec des extrêmes allant de 14 à 40. Six patientes ont accouché. Il s´agissait d´un accouchement prématuré dans quatre cas (4/11) dont deux prématurités induites pour SFA (souffrance fœtale aigüe) et deux RPM (rupture prématuré des membranes) qui étaient entré spontanément en travail. Deux patientes avaient accouché à terme. Parmi les 11 patientes, cinq patientes (5/11) étaient rentrées chez elles. Le diagnostic d´une menace d´accouchement prématuré légère était retenu chez deux parmi les cinq patientes. Le diagnostic d´un faux travail était retenu chez deux patientes et une menace d´avortement tardive à 14 SA jugulée par le simple repos chez une patiente. La voie d´accouchement était une césarienne dans cinq cas. Nous avons eu deux transferts en néonatologie avec un score d´Apgar à 5/7 dans les deux cas.

**Traitements et évolution:** deux patientes étaient prises en charge ultérieurement en réanimation médicale pour syndrome de détresse respiratoire aigüe (SDRA). La durée d´hospitalisation en réanimation était de 8 jours. Ces deux patientes ont nécessité l´Optiflow et l´amoxicilline + acide clavulanique pour prévention des surinfections. Aucune patiente n´avait nécessité de ventilation mécanique. Dans les neuf autres cas, le traitement symptomatique et l´antibiothérapie avait suffi. L´auto-isolement de ces patients était poursuivi à domicile pendant 14 jours. Les deux menaces d´accouchement prématuré légères avaient été jugulées par chlorhydrate de nicardipine en ambulatoire. En addition au traitement symptomatique et antibiotique, un cas de menace d´avortement tardive avait nécessité un simple repos.

**Transmission materno-fœtale:** nous n´avons eu aucune transmission materno-fœtale. Nous avons testé la présence du virus dans de gorge néonataux. Uniquement, les deux nouveau-nés transférés en néonatologie ont bénéficié de cet examen.

**Allaitement:** nous avons encouragé l´allaitement maternel chez 9 de nos patientes. Aucune transmission néonatale n´a été détecté. Les deux patientes transférées en réanimation n´ont pas pu allaiter leurs enfants. Elles ont bénéficié d´une prise en charge en néonatologie.

**Décès:** nous n´avons rapporté aucun cas de décès pendant la durée de notre étude.

## Discussion

**Les points forts:** il n´existe pas assez de travaux sur ce sujet. C´est une pathologie nouvelle et nous n´avons pas assez de recul. Alors que l´organisation de compagne de vaccination s´organise en occident. Nous, en Afrique, nos estimations, selon le ministère de santé Tunisienne, seraient l´apport du vaccin vers le mois d´Avril 2021. D´où l´utilité de continuer à étudier cette pathologie et en conclure des lignes de conduite afin de faire face à cette deuxième vague.

**Limites de notre étude:** il serait judicieux d´avoir de plus grande série, multicentrique, à l´échelle nationale, afin d´avoir des résultats plus significatifs.

**Résumé des résultats:** onze patientes ont été colligées. La moyenne d´âge est de 31,18 ans. Six patientes étaient suivies pour une maladie chronique. Le diabète gestationnel était la comorbidité la plus fréquente, présente chez cinq patientes. Un contact avec un cas suspect ou confirmé de COVID-19 était identifié chez 6 patientes .La durée moyenne d´incubation du virus était de 5.89 jours. Malgré les moyens de protection, nous avons eu la contamination de 12 personnes travaillant au service. Le TAG moyen était de 33,36 semaines d´aménorrhées. La voie d´accouchement était une césarienne dans cinq cas. Le délai moyen entre l´installation des symptômes et l´hospitalisation était de 6,55 jours. Les symptômes les plus fréquemment rapportés étaient la fièvre chez toutes les patientes, la toux sèche, la dyspnée et la céphalée chez neuf patientes. La CRP était élevée dans neuf cas .Toutes les patientes ont eu un écouvillonnage naso-pharyngé adressé pour recherche du coronavirus par PCR. Deux patientes étaient prises en charge ultérieurement en réanimation médicale pour un tableau de syndrome de détresse respiratoire aigüe (SDRA). Dans les 9 autres cas, le traitement symptomatique et l´antibiothérapie avaient suffi et les patientes sont retournées à leur domicile. Nous n´avons eu aucune transmission materno-fœtale et aucun cas de décès. L´allaitement ne semble pas influencer cette transmission.

Le virus semble transmis principalement par les gouttelettes [8]. Le port du masque dans les espaces clos et en extérieur dans certains lieux est devenu obligatoire en Tunisie avec une nouvelle saturation du système hospitalier et une augmentation des décès. Dans notre étude, un contact avec un cas suspect ou confirmé de COVID-19 était identifié chez 6 patientes. Il s´agissait d´un membre de la famille dans quatre cas. Malgré les moyens de protection (Le masque de protection respiratoire individuelle de type FFP2, visière, gants et blouse jetables pour tous les soins) six patientes parmi les 11 seraient responsables de la contamination de 12 personnes travaillant au service. Les données de la littérature suggèrent que la période d´incubation serait d´environ 5 jours (entre 2 et 14 jours) [8]. La période de contagiosité pourrait précéder les symptômes [9]. Dans notre étude, la durée moyenne d´incubation du virus était de 5.89 jours. Zhu *et al*. ont rapporté rétrospectivement le suivi de neuf mères dans cinq hôpitaux du Hubei [10]. Quatre ont présenté des symptômes dans les quatre jours avant l´accouchement, deux le jour de l´accouchement et trois par la suite. Dans la littérature, la majorité des personnes (80%) [11] infectées par le SARS-CoV-2 et présentant des symptômes n´ont eu qu´une légère rhinite ou un syndrome grippal léger ou modéré. Des symptômes plus graves ont également été décrits (16 à 32%), présents chez les patients présentant une immunodépression ou des comorbidités et les femmes enceintes [12].

Les symptômes les plus courants au début de la maladie étaient la fièvre (98%) et la toux (76%) [8]. Les symptômes les plus fréquemment rapportés dans notre enquête étaient la fièvre chez toutes les patientes, la toux sèche, la dyspnée et la céphalée chez 9/11 patientes. Dans l´étude de Chen *et al*. [5], il y avait neuf patientes enceintes au troisième trimestre testées positives pour le SARS-CoV-2. Sept femmes ont présenté de la fièvre, quatre de la toux, trois des myalgies, deux une odynophagie et deux des malaises. Selon Chen *et al*. [5], cinq présentaient une lymphopénie, 3 des perturbations du bilan hépatique. Dans notre étude, les explorations biologiques ont objectivé deux bilans hépatiques perturbés à type de cytolyse hépatique estimée à 2-3 fois la normale. Une ascension de la CRP constatée chez neuf patientes, et une numération formule sanguine correcte chez toutes les patientes. Liu *et al*. [12] ont publié une série de 13 femmes enceintes infectées sans comorbidités. Le symptôme principal était la fièvre (77%). Vingt-trois pour cent présentaient une dyspnée. Trois patientes ont pu rentrer à leur domicile sans complication connue. Dix (77%) ont été césarisées, en particulier pour anomalies du RCF (rythme cardiaque fœtal) (3 patientes), rupture de la poche des eaux (1 patiente) et sauvetage maternel dans un contexte de mort in utero, la patiente ayant développé un SDRA, une insuffisance hépatique, une insuffisance rénale aiguë et un choc, nécessitant une ECMO (l'acronyme anglais de extracorporeal membrane oxygenation) à 34 SA.

Dans notre série, six patientes ont accouché parmi les onze. Il s´agissait d´un accouchement prématuré dans quatre cas. Cinq patientes sont rentrées chez elles. Le diagnostic d´une menace d´accouchement prématuré légère était retenu chez deux parmi les cinq patientes. Le diagnostic d´un faux travail était retenu chez deux patientes, une menace d´avortement à 14 SA jugulée par le simple repos chez une patiente. Deux patientes ont nécessité un transfert en réanimation dans un tableau de syndrome de détresse respiratoire aigüe (SDRA). La voie d´accouchement privilégiée est la césarienne. La prise en charge thérapeutique des patients atteints du COVID-19 n´est pas encore codifiée. Aucun traitement antiviral spécifique n´a démontré son efficacité. Cela renforce l´importance à accorder aux traitements symptomatiques. Dans notre étude, il n´y avait aucun cas de transmission materno-fœtale. Les connaissances à l´heure actuelle sont très limitées sur le sujet. Dans l´étude de Chen *et al*. [5] la transmission materno-fœtale a été évaluée en testant la présence du virus dans des échantillons de liquide amniotique, de sang de cordon et d´écouvillons de gorge néonataux chez 6 des 9 enfants. Tous les prélèvements réalisés étaient négatifs. Liu *et al*. ne trouvent pas non plus de nouveau-nés infectés [12]. L´analyse de ces petits groupes de patientes suggère qu´à l´heure actuelle il n´y a aucune preuve d´infection materno-fœtale.

Nous n´avons eu aucune transmission materno-fœtale. Nous avons testé la présence du virus dans de gorge néonataux. Aucune étude publiée spécifiquement sur la transmission du SRAS-CoV 2 lors de l´allaitement n´a été identifiée dans la littérature scientifique [13]. L´OMS et l´UNICEF s´entendent sur l´importance de continuer l´allaitement, même en cas d´infection par le SARS-CoV-2. Ces organisations insistent sur le fait que les bienfaits de l´allaitement sont grands et que le risque de transmettre le virus par le lait est faible puisqu´il s´agit d´un virus respiratoire [14]. Le taux de létalité est estimé à 1% [15]. Nous n´avons pas eu de décès dans notre série. La priorité doit être donnée à la réalisation d´études de haut niveau de preuve. Il faut encourager une attention particulière aux molécules dont les données précliniques sont prometteuses. Pfizer et BioNTech vont développer conjointement un vaccin contre le COVID-19, dans un premier temps aux États-Unis et en Europe, avant de renforcer les capacités de production afin de garantir un approvisionnement à l´échelle mondiale [16]. Un dispositif mis en place par GAVI, l´alliance pour les vaccins et l´Organisation Mondiale de la Santé (OMS) pour acheter et distribuer les vaccins anti COVID-19 aux pays en développement a permis de réunir plus de deux milliards de dollars pour un engagement dit de « garantie de marché » [17]. Ces fonds permettront à ces agences onusiennes d´acheter une première commande d´un milliard de doses de vaccins pour 92 pays éligibles [17]. Nous, africains, nous devront faire face encore à ce virus, renforçant la compréhension de la maladie et se basant sur le traitement symptomatique.

## Conclusion

Les symptômes les plus fréquemment rapportés étaient la fièvre chez toutes les patientes, en deuxième lieu la toux sèche, la dyspnée et la céphalée. La voie d´accouchement la lus privilégiée était la césarienne. Il n´y a pas eu de transmission materno-fœtale, ni de transmission au cours de l´allaitement. En général le traitement symptomatique et à base d´antibiothérapie avait suffi. Nous faisons actuellement face à la deuxième vague du virus, avec l´arrivée de l´hiver, nos structures sanitaires seront mises à rude épreuve. L´espoir de l´arrivée d´un vaccin sera plus tardif dans notre continent, nos efforts devront se baser sur une meilleure connaissance clinique de ce nouveau coronavirus et adapter une conduite en fonction.

### Etat des connaissances sur le sujet

- Les études s´intéressant à la femme enceinte ne sont pas nombreuses. La symptomatologie théoriquement peut être plus grave que la population générale.

### Contribution de notre étude à la connaissance

- Il s´agit à notre connaissance de la première étude tunisienne s´intéressant au COVID-19 chez la femme enceinte.
